# Do Beliefs About Whether Others Can See Modulate Social Seeking in Autism?

**DOI:** 10.1007/s10803-018-3760-1

**Published:** 2018-10-04

**Authors:** Roser Cañigueral, Antonia F. de C. Hamilton

**Affiliations:** Present Address: UCL Institute of Cognitive Neuroscience, University College of London, Alexandra House, 17 Queen Square, London, WC1N 3AZ UK

**Keywords:** Autism, Social motivation, Perceptual mentalizing, Eye gaze

## Abstract

Autistic people process gaze differently than typical people, but it is not yet clear if these differences lie in the processing of eye-shapes or the belief in whether others can see (perceptual mentalizing). We aimed to investigate whether these two models of gaze processing modulate social seeking in typical and autistic adults. We measured preferences of participants to view videos of an actress with visible or hidden eyes, who can or cannot see out. While typical participants preferred videos where the actress can see through and has visible eyes, autistic people showed no preference for these videos. These findings are discussed in the context of perceptual mentalizing and the social motivation theory of autism.

**Electronic supplementary material:**

The online version of this article (10.1007/s10803-018-3760-1) contains supplementary material, which is available to authorized users.

## Introduction

Eye gaze is a powerful and compelling social cue. It can be rapidly detected (Senju and Hasegawa 2005) allowing discrimination of complex emotions (Baron-Cohen et al. 1997) and intentions (Senju and Johnson 2009a). Direct gaze also signals that another person can see out, and this belief in seeing something has been linked to mentalizing (Teufel et al. 2010a). A variety of studies suggest that comprehension of gaze and responses to gaze may be different in autistic people (Senju and Johnson 2009a). Recently, it has been found that typical adults prefer viewing faces with direct gaze to those with averted gaze, while no such preference is seen in autism (Dubey et al. 2015). However, it is not yet clear if differences in gaze preference in autism are related to processing the gaze as a physical eye-shape, or to differences in the belief of whether others can see something. The present paper uses a novel paradigm to explore these different possibilities.

### Models of Gaze Processing

Current models of gaze processing suggest two possible neurocognitive responses to the gaze of another person—a basic alerting mechanism and a more elaborate perceptual mentalizing process. The basic alerting mechanism is believed to be implemented by a sub-cortical pathway, whereby an image of a pair of eyes activates the superior colliculus, pulvinar and amygdala (Senju and Johnson 2009b), and directs attention towards the eyes (Senju and Hasegawa 2005). It is thought that this pathway has an evolutionary meaning, since in many non-human species direct gaze signals threat from a predator (Emery 2000). In humans, neuroimaging studies indicate that the subcortical regions modulate, in turn, the recruitment of the social brain network (e.g. superior temporal sulcus, medial prefrontal cortex), which modulates the processing of contextual social information (Senju and Johnson 2009b). Overall, the subcortical mechanism operates rapidly and seems to function in the same way for all stimuli which match the basic features of a pair of eyes. Thus, we could describe this attentional gaze processing as being driven by an ‘eye-shape’, regardless of whether that is a photo, a schematic drawing or a live person.

More recently, research has also begun to uncover how people process gaze as a form of perceptual mentalizing. It is important to distinguish perceptual mentalizing from the audience effect. Perceptual mentalizing is the attribution of perceptual states to other people about whether they can or cannot see *something* (Teufel et al. 2010a). Instead, the audience effect refers to changes in behaviour that happen under the belief that other people can specifically see *me* (Hamilton and Lind 2016). Substantial research has investigated the audience effect, showing that the belief in being seen modulates emotional arousal (Myllyneva and Hietanen 2015), eye gaze directed at the observer (Gobel et al. 2015; Laidlaw et al. 2011), and prosocial behaviour (Izuma et al. 2009). However, only few studies have looked at how perceptual mentalizing influences social information processing.

For instance, Teufel et al. (2010b) found that gaze cueing effects are driven by perceptual mentalizing. Participants saw videos of an actor wearing obscured goggles with different coloured frames, and were told that one pair of goggles was transparent whereas the other was opaque; participants were also told that the videos were live streamed from an adjacent room. Results showed that the gaze cueing effect was greater in valid trials (i.e. trials where actor’s gaze and target are on the same side) only for the transparent goggles. This suggests that the gaze cueing effect was strongly influenced by the attribution of meaningful mental states to the confederate, and that this only happened when the confederate could see through the goggles (see Nuku and Bekkering 2008 for a similar study).

In another study, Furlanetto et al. (2016) showed that perceptual mentalizing also influences visual perspective taking. In each trial participants first saw the words ‘YOU’ or ‘SHE/HE’ on the screen, then a digit, and finally an avatar looking towards the right or left wall of a room (created with a virtual reality software). Each wall had a number of dots that ranged from 0 to 2. Participants had to judge whether the digit was consistent with the number of dots that themselves (‘YOU’) or the avatar (‘SHE/HE’) could see, respectively. Crucially, this task was combined with the goggles paradigm designed by Teufel et al. (2010b): the avatar was wearing obscured goggles that participants believed to be either transparent or opaque. They found that reaction times were slower when the avatar was wearing transparent goggles, suggesting that participants took the perspective of the avatar only when they knew the avatar could see the dots through the goggles.

Thus, these studies show that perceptual mentalizing itself modulates how we process social information from eye gaze. Here, we aim to test the relationship between perceptual mentalizing and social motivation, and whether this relationship is different in autism.

### Gaze and Social Interaction in Autism

People with the Autism Spectrum Condition (ASC) show persistent difficulties in social interactions (DSM-5; Diagnostic and Statistical Manual of Mental Disorders 5th Ed. 2013) including the processing of eye gaze. Eye tracking studies suggest autistic people make fewer fixations on the eyes of actors in a movie (Klin et al. 2002), though this effect is reduced for static photos of faces (Chevallier et al. 2015). They have also difficulties in understanding the social meaning of gaze duration and gaze direction, and show abnormal recruitment of brain areas that process this information (Georgescu et al. 2013; Senju et al. 2005). At the same time, there is evidence suggesting that autistic people might be less susceptible to the belief in being seen (audience effect): for instance, they do not seem to increase prosocial behaviour when other people are observing them (Cage et al. 2013; Izuma et al. 2011). However, no previous research has yet addressed perceptual mentalizing in autism. Thus, it is not yet clear if differences in responsiveness to gaze in autism are due to changes in the subcortical gaze-attention mechanism, or due to differences in perceptual mentalizing about whether others can see something. The present paper investigates this issue, in relation to the measurement of motivation.

Recently, it has been suggested that typical adults find social stimuli and social interactions inherently rewarding, possessing an inbuilt social motivation, but that this motivation might be reduced in autism (Chevallier et al. 2012). For instance, autistic participants self-report diminished enjoyment in social situations when compared to typical ones (Chevallier et al. 2011). At the neural level, they activate reward-related brain areas (e.g. ventral striatum) significantly less than typical people, specifically in response to social reward (Scott-Van Zeeland et al. 2010; see also; Delmonte et al. 2012 and; Kohls et al. 2013). Chevallier et al. (2012) distinguish three levels of social motivation. First, *social orienting* happens when we give attentional priority to social stimuli rather than non-social; second, there is *social seeking* when we make an effort to get social stimuli because we like them (i.e. we want them because we find them inherently rewarding); finally, *social maintaining* refers to the development of strategies to enhance relationships with others (e.g. be viewed as likeable, cooperative, etc.). For the scope of the present study we will focus on social seeking.

A number of studies have found ways to measure social seeking behaviour. For example, people will sacrifice a small monetary reward to view a genuine smile (Shore and Heerey 2011), and will press a key repeatedly to see attractive people (Hayden et al. 2007). A new measure of social seeking behaviour has been recently developed, which attempts to quantify how much effort people will make to seek out social stimuli. In this Choose-A-Movie (CAM) paradigm, participants are presented on each trial with two coloured boxes and know that (for example) the orange box contains movies of people, while the green box contains movies of household objects. They can chose which box to open and thus which movie category to watch on each trial. To encourage careful decision making, on each trial there are a number of locks on each box (one, two or three locks), and to open a box with three locks participants must press a key and wait for that lock to open three times. This imposes a small but noticeable effort cost on that box, compared to a box with one lock. Thus, participants must trade-off their preference to view a particular movie against the effort involved in viewing it. Results show that they do just that (Dubey et al. 2015). More critically, the data show that typical adults prefer to view direct gaze video-clips to averted gaze video-clips and object video-clips. Conversely, autistic individuals prefer object clips to direct or averted gaze, and have a weak preference for averted gaze over direct gaze.

However, it is not yet clear why autistic people show differences in their motivation to view direct gaze stimuli. Many differences in gaze processing have been reported in autism, such as gaze comprehension (Baron-Cohen et al. 1997) and attentional cueing from gaze (Senju and Johnson 2009a). These effects could be explained by differences in a basic sub-cortical mechanism sensitive to eye-shapes. Autistic individuals also show differences when attributing mental states to others (Baron-Cohen et al. 1985; Senju et al. 2009), and seem to be less sensitive to the presence of other people observing them (Cage et al. 2013; Izuma et al. 2011). This suggests that differences in response to direct gaze might reflect differences in perceptual mentalizing capacities. In the present study, we contrast these two hypotheses in the context of social motivation. Our task also allows us to distinguish whether differences in social motivation are due to active avoidance of eye gaze and social information or passive omission of these cues (Corden et al. 2008; Senju and Johnson 2009a).

### The Present Study

The present study aimed to dissociate responses to eye-shapes, presumably processed by a subcortical mechanism (Senju and Johnson 2009a, b), and responses to the belief that another person can see something, presumably related to perceptual mentalizing (Teufel et al. 2010a). To test responses to eye-shapes, we contrasted videos of an actress with empty sunglasses (no lenses) to videos of the actress wearing normal sunglasses. To test responses to the belief that others can see, we adopted the manipulation of Teufel et al. (2010b), where participants believed the actress can see through one set of sunglasses (normal sunglasses, e.g. those with blue frames) but cannot see through the other (opaque sunglasses, e.g. those with red frames). We counterbalanced which frame colour was linked to each belief (can see or cannot see) across participants. To complete our 2 × 2 factorial design, we created videos of the actress wearing sunglasses with paper eyes glued over the lenses—typical eye-shapes are present in this manipulation, but the actress cannot see through. This set of stimuli gave a complete 2 × 2 factorial design with levels Belief in seeing (Belief, B+ and B−) and Eye-shape (Eyes, E+ and E−); sample stimuli from each cell are shown in Table 1.

**Table 1:** Factorial design used for the video categories and sample screenshots of the video-clips

	Eye-shape (E)	
	Visible (+)	Hidden (−)
Belief in being seen (B)		
Yes (+)	Empty sunglasses (B+E+)	Normal sunglasses (B+E−)
No (−)	Paper-eyes sunglasses (B−E+)	Opaque sunglasses (B−E−)

Taking these four social video-clips, we used the Chose-A-Movie task to measure which movie participants preferred to view. Expanding on the design by Dubey et al. (2015), we gave participants a choice of all four movies on each trial, with variable numbers of locks to manipulate effort. Our primary outcome measure was the percentage of trials on which participants chose each movie. Note that we did not attempt to persuade participants that these videos represented a live-feed of another person from another room. We took this decision for three reasons. First, we wanted to specifically test the effect of perceptual mentalizing (belief that someone can see *something*) on social motivation. Second, we wanted to remain close to the procedure of Dubey et al. (2015), where there was no live-feed manipulation. Third, it is not very plausible to have a live-feed of the same person wearing multiple different sets of sunglasses at very short notice. Thus, our experimental design addresses perceptual mentalizing in a non-interactive way: participants know that they are not in an online interaction with the actress.

Based on our hypotheses, we can make several predictions. First, if typical participants prefer attributing perceptual mental states because they have no difficulties in further social information processing, they should choose to view stimuli with empty and normal sunglasses (B+ conditions, actress can see through) over stimuli with paper-eyes and opaque sunglasses (B− conditions, actress cannot see through). Second, if autistic people show reduced social motivation because they are not sensible to eye-shapes, they will show no difference in preference for E+ and E− conditions. However, if autistic individuals actively avoid eye-shapes, they should prefer videos with normal and opaque sunglasses (E− conditions, eyes not visible) to videos with empty and paper-eyes sunglasses (E+ conditions, eyes visible). Third, if autistic people show reduced social motivation due to difficulties in attributing a perceptual mental state to the actress, they should show no difference in preference for B+ and B− conditions. If they attribute perceptual mental states but are less motivated to engage because of difficulties in further processing of this or other social information, they should prefer videos with opaque and paper-eyes sunglasses (B− conditions) to videos of empty and normal sunglasses (B+ conditions). Systematic group differences should be reflected in Group × belief and group × eyes interactions. Because the paper-eyes sunglasses are a slightly odd stimulus with low ecological validity, the strongest test of the claim that beliefs about whether someone can see impact on social motivation comes from examining the conditions where participants cannot see the eyes of the actress but their belief about whether or not she can see through is manipulated. Thus, the Group X Belief interaction was also examined including only the E− conditions, that is, normal sunglasses (B+E−) and opaque sunglasses (B−E−).

## Methods

### Participants

A group of 25 typical adults and 27 adults with ASC participated in this experiment. Previous studies (Dubey et al. 2015) show that this sample size is suitable to detect differences between two groups. Table 2 shows that the participants included in the analyses (i.e. after removal of outliers; sample of 25 typical and 24 ASC participants) were matched on age, gender, handedness and Intelligence Quotient (IQ; Wechsler Adult Intelligence Scale, WAIS-III UK, Wechsler 1999a, b; or Wechsler Abbreviated Scale of Intelligence, WASI-II); note that the ASC group was high functioning, which means that their IQ is higher than 80: since both groups were matched, the typical group also had high IQ on average. The two groups differed on Autism Quotient (AQ; Baron-Cohen et al. 2001). All participants were recruited using an autism database at the author’s institution. Recruitment of ASC participants was based on diagnosis from an independent clinician. Routine diagnostic procedures include the Autism Diagnostic Observation Schedule (ADOS; Lord et al. 2000) and the Autism Diagnostic Interview-Revised (ADI-R; Lord et al. 1994), among others. ASC participants included in the analyses were diagnosed as either Asperger’s Syndrome (17), Autism (6) or Autism Spectrum Disorder (1). They were also tested on module 4 of the ADOS by a trained researcher: seven participants met the ADOS classification for Autism; ten for Autism Spectrum; seven did not meet the classification for any of them, but all seven participants had a clear diagnosis from an independent clinician, and reached the cut-off for autism spectrum on either the communication or reciprocal social interaction subscale. They gave written informed consent before doing the experiment and were compensated for their time and travel expenses. All procedures were approved by the local Research Ethics Committee and were in accordance with the Declaration of Helsinki and APA ethical standards.

**Table 2:** Comparison of the typical and autistic (ASC) samples that were included in the analyses

	Typical (N = 25)		ASC (N = 24)		*t*-test
	Mean (SD)	Range	Mean (SD)	Range	*p*-value
Age	32.28 (10.26)	19–58	33.42 (10.18)	19–52	0.70
Gender	5 F, 20 M	–	1 F, 23 M	–	–
Handedness	2 L, 23 R	–	3 L, 21 R	–	–
IQ: full-scale	115.52 (13.61)	93–149	116.42 (13.35)	86–152	0.82
IQ: verbal	116.32 (13.32)	96–150	118.13 (15.10)	91–155	0.66
IQ: performance	111.00 (14.36)	80–136	111.13 (13.35)	80–132	0.97
AQ	15.60 (4.66)	6–22	31.00 (10.27)	10–46	0.00***
ADOS: total	–	–	8.92 (3.41)	4–17	–
ADOS: communication	–	–	2.58 (1.64)	0–6	–
ADOS: social interaction	–	–	6.33 (2.08)	4–11	–

*F* female, *M* male, *L* left, *R* right

*p < 0.05, **p < 0.01 and ***p < 0.001

### Stimuli

Four different types of video-clips were created. These video-clips corresponded to the four categories of a 2-by-2 factorial design with factors Belief (B) and Eyes (E) (see Table 1), which were determined by the type of sunglasses the actress on the video-clip was wearing. The four categories were the following: B+E+ (belief in seeing through and eyes visible: empty sunglasses), B−E+ (no belief in seeing through but eyes visible: paper-eyes sunglasses), B+E− (belief in seeing through but eyes not visible: normal sunglasses) and B−E− (no belief in seeing through and eyes not visible: opaque sunglasses). Empty sunglasses had black frames and no lenses; paper-eyes sunglasses had black frames and a picture of an eye glued over each lens; normal and opaque sunglasses had either blue or red frames (counterbalanced across participants) and mirrored lenses (so that participants could not see the eyes of the actress); opaque sunglasses also had a black paper glued behind each lens.

During the filming session, we initially recorded eight different actors (four female, four male). They sat in front of a blue background and the camera was prepared to depict a medium close shot. They were instructed to first look down and, upon hearing the experimenter call their name, look up directly to the camera and smile as if they were greeting a friend. They were recorded while wearing empty sunglasses, paper-eyes sunglasses, and normal sunglasses (with both red and blue frames). Since the lenses of the sunglasses were mirrored, the videos with normal sunglasses were also used for the opaque sunglasses condition: this ensured that there were no perceptual differences in the face between these two conditions. The final video-clips had a duration of 3 s, and were saved at 600 × 450 pixels.

All 32 videos (eight possible actors for each of the four video categories) were rated on trustworthiness, friendliness and genuineness of the greeting (see Supplementary Materials S1). We found that one of the female actresses received higher ratings than the other actors, for all four video categories. Her performance matched our requirements for these stimuli, since we needed to ensure that social seeking was not affected by the actress being too neutral on the facial expression. Thus, we decided to only use this actress in the stimuli.

**Fig. 1:**
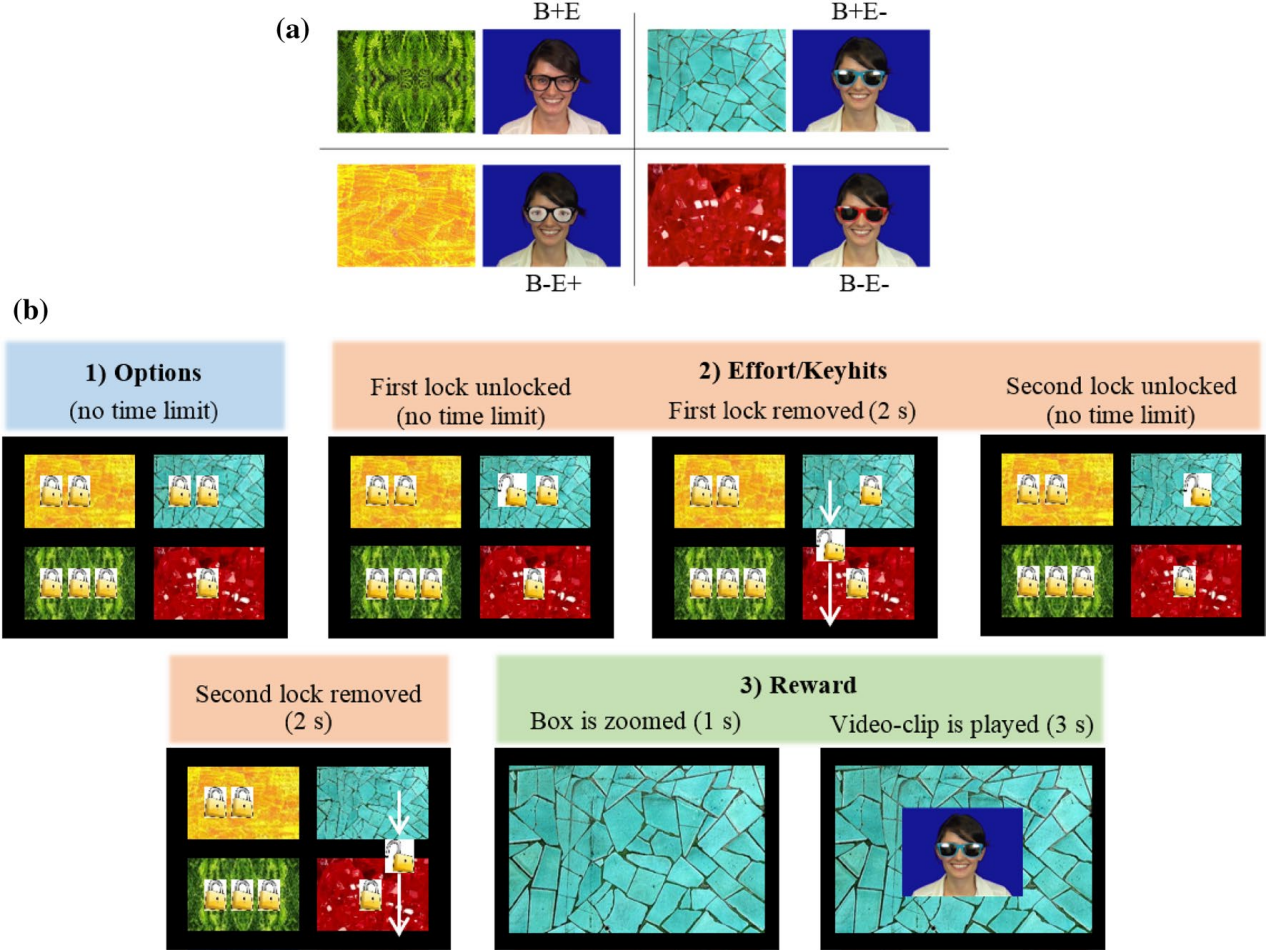
**a** Association between patterned boxes and video-clip categories. **b** Sample trial where the participant chooses B+E− condition (normal sunglasses): (1) the four options are first displayed; (2) the participant chooses which box to open by doing the required key hits; (3) the unlocked box is zoomed and the corresponding video-clip is displayed as a reward

In addition to the video-clips, four abstract coloured patterns were used as cues for the four video categories (Fig. 1a). Patterns associated with empty (green pattern) and paper-eyes (yellow pattern) sunglasses were the same for all participants. Patterns associated with normal and opaque sunglasses were counterbalanced according to the counterbalancing of the frame colour of the sunglasses (red and blue patterns).

### Choose-a-Movie (CAM) Task Design

The choose-a-movie task design was coded with MATLAB (8.5, MathWorks 2015) and Cogent Graphics. Similar to Dubey et al. (2015), participants first learnt that each video-clip category was linked to one particular patterned box (see Fig. 1a). Then, on each trial (see Fig. 1b for a sample trial), participants saw four patterned boxes on the screen, and each box could have 1, 2 or 3 locks. All possible combinations of locks (3) and video categories (4) appeared across trials, and each video category randomly appeared in one of the four positions on the screen. Participants unlocked the boxes by pressing a specific key on the numeric keypad in front of them. The key they were required to press was linked to the position of the box (one for bottom–left, three for bottom–right, seven for top–left, nine for top–right), and they had to press the key as many times as number of locks the box had (e.g. if the box had three locks, they would press the key three times). Thus, on each trial participants had to choose their preferred video category in trade-off with effort (i.e. number of locks or keyhits). When a box was unlocked, the video-clip with the actress wearing the corresponding type of glasses was displayed as a reward.

### Procedure

Participants came to the lab to perform the experiment as part of a Research Day. They sat in a chair approximately 1 m from the projector screen, and between them and the screen there was a table with the numeric keypad. The experimenter first showed the four types of sunglasses to the participants in order to manipulate their beliefs. For each type of sunglasses, the experimenter first named the sunglasses and then described whether a person would be able to see through them or not. The sunglasses were always presented in the same order: empty sunglasses, paper-eyes sunglasses, normal sunglasses and opaque sunglasses. However, for half of the participants the normal sunglasses had blue frames and the opaque sunglasses had red frames, whereas for the other half it was the opposite. Participants tried all the sunglasses and left them on the table. In front of each type of sunglasses the experimenter placed a label with the name of the sunglasses. Participants then started the learning phase, during which they learnt that each video category was linked to one particular patterned box. In case they forgot the link between patterns and video category, the background of the label in front of each type of glasses matched the corresponding pattern for that category. During the learning phase participants completed eight trials in order to practise how to unlock boxes. Then participants performed the test phase, during which they completed 81 trials (i.e. all possible combinations of three locks and four video categories) across three blocks (27 trials in each block). Because participants originally performed a mimicry task interspersed between the CAM task blocks, the overall duration of the experiment was approximately 45 min, from which 20 min where devoted to the CAM task (the results from the mimicry task will be reported elsewhere). In the end of the task participants completed a post-test questionnaire in order to check that they understood the meaning of each type of glasses (see Supplementary Materials S2). Only two typical participants and three ASC participants gave a wrong answer in one of the questions. The two typical participants responded wrongly the questions about normal (red in the questionnaire) and opaque (blue in the questionnaire) sunglasses, respectively. The three ASC participants responded wrongly the questions about paper-eyes (fake-eyes in the questionnaire), normal (blue in the questionnaire) and opaque (red in the questionnaire) sunglasses, respectively. Removal of these participants did not affect the results so they were all included in the statistical analysis. Data on ADOS scores, IQ scores and AQ scores for all participants was available from previous testing sessions at the author’s institution.

### Data Analysis

The primary outcome measure for each participant was the percentage of trials on which each video was chosen. There were four possible video-clip categories, so 25% was considered chance level. Three outliers within the autistic sample were removed, since their mean percentage of choices for one of the conditions was above 3 SDs from the overall mean. In particular, each of these three participants had chosen the same condition in more than 95% of the trials (these conditions were empty sunglasses, paper-eyes sunglasses and normal sunglasses), which suggests that their choice may based on a particular preference for the colour or pattern associated with that video category. Statistical analyses were performed for both the full sample and the final sample excluding outliers (25 typical and 24 autistic participants; see Table 2). A three-way repeated measures ANOVA was performed. Belief in seeing (B+ and B−) and Eye-shape (E+ and E−) were used as within-subjects factors, Group (typical and autism) as between-subjects factor, and the measure percentage of choices as dependent variable. However, to test and interpret our hypotheses we focused on two-way interactions between Belief X Eyes, Belief X Group and Eyes X Group. The Belief X Group interaction was also examined including only the E− conditions, that is, normal sunglasses (B+E−) and opaque sunglasses (B−E−). Post-hoc pairwise comparisons using Bonferroni’s adjustment were also computed. Since the analyses for the full and final sample showed the same pattern of results, here we only report the results for the final sample.

When analysing choice preferences it is important to take into account how the choice of a particular video category was affected by the relative effort required to watch that video. The algorithm implemented in Dubey et al. (2015) can only be used in designs with two options, but ours has four options. Instead, we developed an algorithm that expanded upon this approach, and that is suitable for designs with four options. For each participant this algorithm computes a set of values (one for each video category) that best explains the choices, taking into account the number of locks present on each trial (1, 2 or 3). We call these values ‘choice scores’. A more detailed description of how the choice score is computed, together with the Matlab script that we used can be found in Supplementary Materials (S3.1). Data inspection using the choice score revealed that the model failed to optimise the values for seven participants, which were excluded from the statistical analysis (two outliers in the typical group and five outliers in the ASC group; but only three outliers in the ASC group were found when using the percentage of choices). The pattern of results using the choice score and the percentage of choices was the same. This indicates that preference for a particular video category, rather than the number of locks on the box, was driving the choices of participants. Since in the analysis with the percentage of choices we can include four extra participants, and the pattern of results is similar in both analysis, here we only report the results for the percentage of choices. The full analysis using choice scores can be found in the Supplementary Materials (S3.2).

Finally, we also tested group differences in the number of locks that were opened on average. We performed a two-way ANOVA with number of locks (1, 2, 3) as within-subjects factor, Group (typical and autism) as between-subjects factor, and proportion of choice of each number of locks as dependent variable (using a sample of 25 typical and 24 autistic participants). Given that the analyses using the percentage of choices (for video categories) and choice score yielded the same pattern of results, we expected no differences between groups regarding the number of locks opened throughout the task. Results showed that this was the case: both groups chose to open one lock more often than two and three locks (see Supplementary Materials, S4). This indicates that both groups put same overall amount of effort to see the videos.

## Results

The percentages of choices were analysed using a three-way repeated measures ANOVA. Results showed that there was no three-way interaction between Belief, Eyes and Group, *F*(1,47) = 0.851, *p* > 0.1, $\eta _{{\text{p}}}^{2}$ = 0.018. This means that preference patterns for particular video categories did not differ between both groups. Thus, a two-way interaction between Belief and Eyes should reveal preference patterns for particular video categories across the whole sample. Moreover, according to our hypotheses, systematic group differences in the motivational value of perceptual states (Belief) and physical cues (Eyes) should be reflected in two-way interactions between Group and Belief, and Group and Eyes. Below we report significant main effects and interactions; descriptives (mean and SD) are given in Table 3, and full statistics and post-hoc tests are given in Table 4 (for all conditions) and Table 5 (for E− conditions only).

**Table 3:** Descriptives for the percentage of choices of each video category

Condition	Group	Mean	SD
Empty sunglasses (B+E+)	Typical	45.4	19.7
	Autism	33.6	11.6
Normal sunglasses (B+E−)	Typical	17.9	14.3
	Autism	20.6	8.31
Paper-eyes sunglasses (B−E+)	Typical	19.6	11.8
	Autism	20.2	7.97
Opaque sunglasses (B−E−)	Typical	17.1	12.7
	Autism	25.1	8.24

**Table 4:** Statistics for the belief × eyes × group repeated measures ANOVA

Belief	Main effect	*F*(1,47) = 23.2; *p* < 0.001***; $\eta _{{\text{p}}}^{2}$ = 0.331
Eyes	Main effect	*F*(1,47) = 16.2; *p* < 0.001***; $\eta _{{\text{p}}}^{2}$ = 0.257
Group	Main effect	*F*(1,47) = 0.067; *p* > 0.1; $\eta _{{\text{p}}}^{2}$ = 0.001
Belief × eyes	Interaction effect	*F*(1,43) = 21.1; *p* < 0.001***; $\eta _{{\text{p}}}^{2}$ = 0.329
	B+: E+ vs. E-	*t*(47) = 5.91; *p* < 0.001***; d_z_ = 0.844
	B−: E+ versus E−	*t*(47) = 0.741; *p* > 0.1; d_z_ = 0.105
	E+: B+ versus B−	*t*(47) = 5.93; *p* < 0.001***; d_z_ = 0.847
	E−: B+ versus B−	*t*(47) = 0.627; *p* > 0.1; d_z_ = 0.089
	B+E+ versus B−E−	*t*(47) = 5.09; *p* < 0.001***; d_z_ = 0.727
	B+E− versus B−E+	*t*(47) = 0.281; *p* > 0.1; d_z_ = 0.040
Belief × group	Interaction effect	*F*(1,47) = 7.67; *p* < 0.01**; $\eta _{{\text{p}}}^{2}$ = 0.140
	B+: typ vs. aut	*t*(47) = 2.83; *p* < 0.01**; d_z_ = 0.404
	B−: typ versus aut	*t*(47) = 2.55; *p* < 0.05*; d_z_ = 0.365
	Typ: B+ versus B−	*t*(47) = 5.42; *p* < 0.001***; d_z_ = 0.774
	Aut: B+ versus B−	*t*(47) = 1.44; *p* > 0.1; d_z_ = 0.205
Eyes × group	Interaction effect	*F*(1,43) = 3.92; *p* = 0.053^+^; n_p_^2^ = 0.077
	E+: typ versus aut	*t*(47) = 2.06; *p* < 0.05*; d_z_ = 0.294
	E−: typ versus aut	*t*(47) = 1.82; *p* = 0.076^+^; d_z_ = 0.259
	Typ: E+ versus E−	*t*(47) = 4.30; *p* < 0.001***; d_z_ = 0.614
	Aut: E+ versus E−	*t*(47) = 1.44; *p* > 0.1; d_z_ = 0.205
Belief × eyes × group	Interaction effect	*F*(1,47) = 0.851; *p* > 0.1; $\eta _{{\text{p}}}^{2}$ = 0.018

*p < 0.05, **p < 0.01 and ***p < 0.001

**Table 5:** Statistics for the belief × group repeated measures ANOVA (E− conditions only)

Belief	Main effect	*F*(1,47) = 0.497; *p* > 0.1; $\eta _{{\text{p}}}^{2}$ = 0.010
Group	Main effect	*F*(1,47) = 4.24; *p* < 0.05*; $\eta _{{\text{p}}}^{2}$ = 0.083
Belief × group	Interaction effect	*F*(1,47) = 3.99; *p* = 0.053^+^; $\eta _{{\text{p}}}^{2}$ = 0.077
	B+: typ versus aut	*t*(47) = 0.176; *p* > 0.1; d_z_ = 0.025
	B−: typ versus aut	*t*(47) = 2.79; *p* < 0.05*; d_z_ = 0.398
	Typ: B+ versus B−	*t*(47) = 0.912; *p* > 0.1; d_z_ = 0.130
	Aut: B+ versus B−	*t*(47) = 1.88; *p* = 0.066^+^; d_z_ = 0.269

*p < 0.05, **p < 0.01 and ***p < 0.001

### Effects of Video Category

**Fig. 2:**
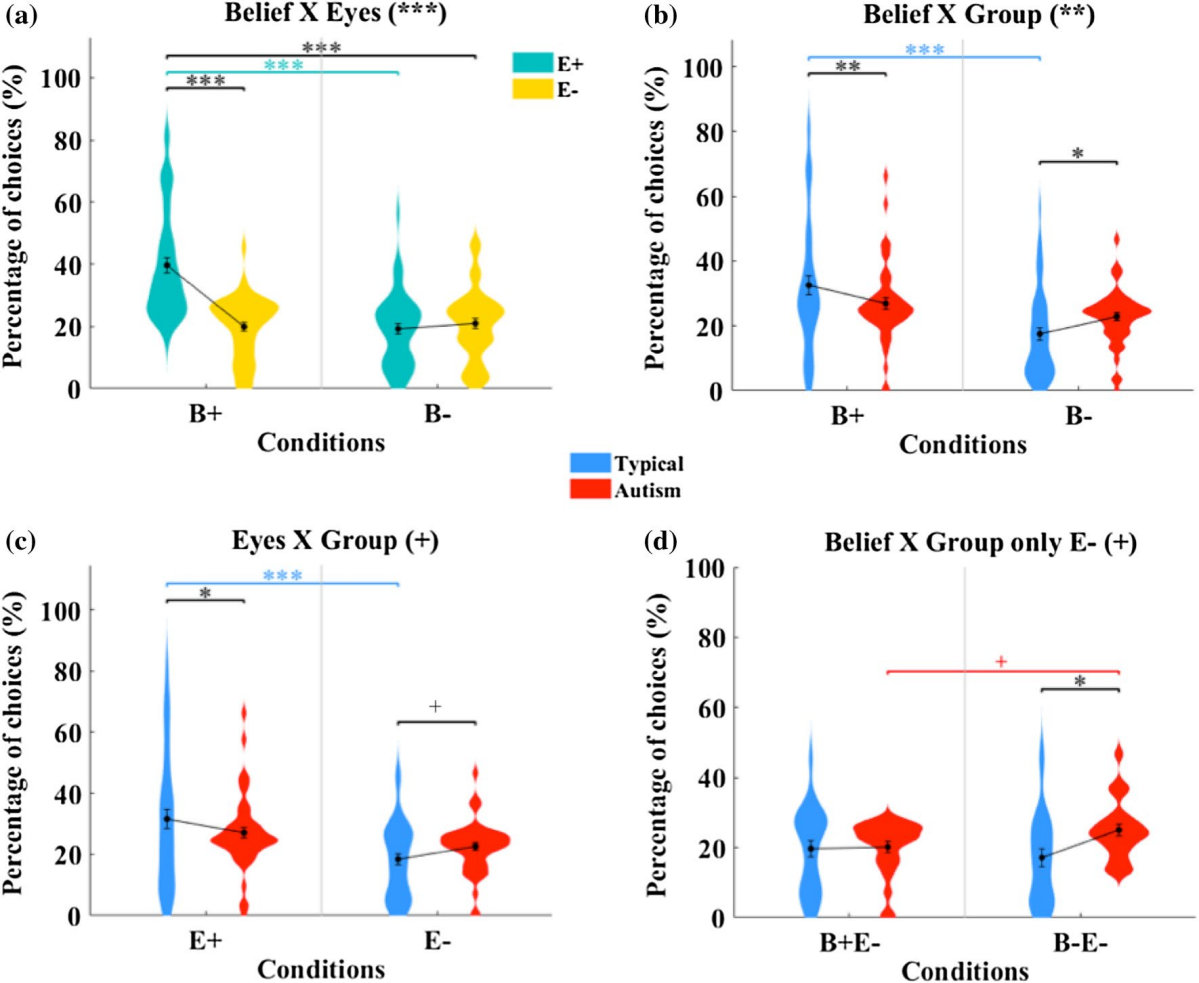
Percentage of choices: mean (filled circle), SE (error bars), and frequency of values (width of distribution). **a** Interaction between Belief and Eyes. **b** Interaction between Belief and Group. **c** Interaction between Eyes and Group. **d** Interaction between Belief and Group for E− conditions only. Asterisks signify difference at ^+^*p* < 0.1, *p < 0.05, **p < 0.01 and ***p < 0.001

Results showed that there was a main effect of Belief, *F*(1,47) = 23.2, *p* < 0.001, $\eta _{{\text{p}}}^{2}$ = 0.331, and Eyes, *F*(1,47) = 16.2, *p* < 0.001, $\eta _{{\text{p}}}^{2}$ = 0.257: B+ and E+ had significantly higher percentage of choices than B− and E−, respectively; however, there was no main effect of Group. There was also a two-way interaction effect between Belief and Eyes, *F*(1,47) = 21.1; *p* < 0.001; $\eta _{{\text{p}}}^{2}$ = 0.329 (Fig. 2a). Post-hoc pairwise comparisons revealed that, taking together both groups (typical and autism), the choice frequency for B+E+ (empty sunglasses) was significantly higher than for B+E− (normal sunglasses), *t*(47) = 5.91, *p* < 0.001, d_z_ = 0.844, B−E+ (paper-eyes sunglasses), *t*(47) = 5.93, *p* < 0.001, d_z_ = 0.847, and B−E− (opaque sunglasses), *t*(47) = 5.09, *p* < 0.001, d_z_ = 0.727. There was no difference between B−E+ and B−E−, *t*(47) > 0.1. These results indicate that participants preferred the videos of the actress with empty sunglasses over any other type of sunglasses.

### Effects of group

There was a two-way interaction effect between Belief and Group, *F*(1,47) = 7.67; *p* < 0.01; $\eta _{{\text{p}}}^{2}$ = 0.140 (Fig. 2b). Post-hoc pairwise comparisons revealed that the choice frequency for B+ was significantly higher than for B− among typical participants, *t*(47) = 5.42, *p* < 0.001, d_z_ = 0.774. When comparing both groups, the frequency of choice of B+ was significantly higher for typical compared to ASC participants, *t*(47) = 2.83, *p* < 0.01, d_z_ = 0.404, whereas for B− it was significantly lower in the typical group, *t*(47) = 2.55, *p* < 0.05, d_z_ = 0.365. This means that typical participants preferred videos where the actress can see through the sunglasses, but this preference was absent in autism. Moreover, typical participants preferred these videos more than autistic participants, whereas the reversed pattern was found for videos where the actress cannot see.

There was a marginal tendency for a two-way interaction effect between Eyes and Group, *F*(1,47) = 3.92, *p* = 0.053, $\eta _{{\text{p}}}^{2}$ = 0.077 (Fig. 2c): similarly to Belief, among typical participants the choice frequency for E+ was significantly higher than for E−, *t*(47) = 4.30, *p* < 0.001, d_z_ = 0.614. The percentage of choices of E+ was significantly higher in the typical compared to the autistic group, *t*(47) = 2.06, *p* < 0.05, d_z_ = 0.294, and a tendency for a higher preference of E− in the autistic group was found when compared to typical participants, *t*(47) = 1.82, *p* = 0.076, d_z_ = 0.259. Despite the absence of strong evidence, these results suggest that typical participants slightly preferred videos with eye-shapes, and that no such preference was found in the autistic group. These findings also indicate that typical participants had stronger preference than the autistic group for videos with eye-shapes, but weaker preference for videos where no eye-shapes were available.

### E− Conditions Only (Normal and Opaque Sunglasses)

The interaction between Belief and Group with the E− conditions was marginally significant, *F*(1,47) = 3.99, *p* = 0.053, $\eta _{{\text{p}}}^{2}$ = 0.077. Post-hoc pairwise comparisons showed that autistic participants preferred B−E− more than the typical group, *t*(47) = 2.79, *p* < 0.05, d_z_ = 0.398. Among typical participants, there was no difference in the percentage of choices between B−E− and B+E−, *t*(47) > 0.1 (Fig. 2d). Among autistic participants, there was a marginal tendency to choose B-E- more often than B+E−, *t*(47) = 1.88, *p* = 0.066, d_z_ = 0.269 (Fig. 2d). Although this evidence is not strong enough to draw decisive conclusions, it suggests that autistic participants may be able to attribute perceptual mental states but have difficulties to process further social information.

## Discussion

The aim of the present study was to investigate how preferences for seeing a physical eye-shape (processed by subcortical gaze-attention mechanisms) and preferences for whether others can see out (processed by perceptual mentalizing) modulate social seeking in typical and autistic adults. All participants preferred videos of a person with full vision (empty sunglasses, B+E+) to other conditions, but this was qualified by a strong interaction effect between Belief and Group, and by a weaker interaction effect between Eyes and Group. While typical participants preferred to watch videos where the actress could see through the sunglasses and eyes were visible over videos where she could not see through and eyes were hidden, autistic participants showed no preference for either video category. Moreover, typical participants preferred B+ and E+ conditions more than autistic participants, but autistic participants preferred B− and E− more than the typical group. Further analyses including only the conditions where eyes are hidden revealed a particular preference for the videos with opaque sunglasses (B−E− condition) in the autistic group. Although some of these results have to be interpreted with caution given the marginal *p*-values and medium effect sizes, we discuss their potential implications in the context of perceptual mentalizing (Teufel et al. 2010a) and the social motivation theory of autism (Chevallier et al. 2012).

### Eye Gaze and Social Seeking in Typical People

Results show that typical participants preferred viewing the videos of a face with eyes that can see through the sunglasses to eyes that cannot see. There is also weak evidence that they preferred eyes that are visible to eyes that are hidden. These results are in line with previous evidence showing that typical people will invest more effort to watch video-clips of a person making eye contact rather than to watch video-clips of a person without eye contact or non-social stimuli (Dubey et al. 2015). It has also been suggested that, in typical natural environments, social interactions are controlled by the interactive processing of beliefs about what others can see and the physical features of eyes (Nuku and Bekkering 2008). Teufel et al. (2010a) also propose that, when processing social information, there is a bidirectional interactive relationship between brain areas processing physical cues and the mentalizing neural system. In the context of our study, it could be that the co-presence of physical and belief cues (i.e. eyes are visible and participants believe that the actress can see through the glasses, B+E+) is more motivating for typical individuals. In line with this, we found that there was a general preference for empty sunglasses when both groups are taken together. However, our study did not find enough evidence to conclude that this preference is exclusive to typical individuals.

Contrary to what we expected, typical participants did not distinguish between the actress wearing normal sunglasses (B+E−) and actress wearing opaque sunglasses (B−E−). This could indicate that typical participants did not use the information they were given about one set of sunglasses being opaque but rather relied on the knowledge that videos were not live streamed. The lack of effect by the opacity of the sunglasses might thus happen because participants were aware that the person in the video could not ever really see them. Teufel et al. (2013) similarly found that, if typical adults do not believe a video is live-streamed, they are not sensitive to an opaque goggles manipulation. Thus, it remains to be seen how much the belief in being seen (i.e. audience effect) matters for social motivation in typical participants. We found that typical participants did distinguish clearly between the empty sunglasses and the paper-eyes sunglasses, where the eye-shape is always present but the belief in whether the other can see something varies. However, it is hard to know if this reflects a true effect of belief: the paper-eyes sunglasses look very different to the other conditions, and some participants reported it was odd or scary to see these videos.

### Eye Gaze and Social Seeking in ASC

When comparing the typical and autistic groups, we found that the latter had weaker preference for videos where the actress can see through the sunglasses (B+) and videos with visible eyes (E+), but stronger preference for videos where the actress cannot see (B−) and videos where eyes are hidden (E−). These findings support those by Dubey et al. (2015), showing that autistic people invest less effort than the typical group to see social stimuli (see also Scott-Van Zeeland et al. 2010 and; Stavropoulos and Carver 2014 for supporting evidence from neuroimaging studies). This was also reflected in an interaction between Belief and Group: while typical participants preferred B+ over B−, autistic participants showed no preference. The interaction between Eyes and Group only yielded a marginal effect with medium effect size (0.077), suggesting that typical participants preferred E+ over E− but there was no preference in the autistic group. If this finding was replicated with stronger evidence, it could proof against theories claiming that autistic people find eye contact aversive (Corden et al. 2008), since it seems they choose B+ and E+ conditions as often as B− and E−, respectively. Rather, autistic individuals might not find eye gaze inherently rewarding, so they passively omit any social information that eyes are carrying.

When only the E− conditions (normal sunglasses and opaque sunglasses) were included in the analyses, there was a marginal interaction between Belief and Group: autistic participants tended to prefer B−E− over B+E−, and they also had stronger preference for B−E− compared to the typical group. These results need careful interpretation, since the marginal p-values and medium effect size of the interaction (0.077) indicate there is not enough evidence to draw decisive conclusions. Nonetheless, they suggest that autistic participants are, to some extent, able to explicitly attribute perceptual states (they relied on the experimenter’s instructions about the sunglasses to make choices), but may find it difficult to further process this or other social information. The absence of beliefs and visible eyes might make the B−E− condition much more comfortable for the autistic participant, who has difficulties in mentalizing (Senju et al. 2009) and in understanding social cues embedded in eye gaze (Baron-Cohen et al. 1997). Previous studies have shown that high-functioning autistic individuals attribute mental states when explicitly prompted, but have difficulties in doing so spontaneously (Senju et al. 2009). It would be interesting to test this when participants need to form beliefs about perceptual states by themselves (i.e. spontaneously): in this context they might find it harder to distinguish between B+E− and B−E−, and might end up choosing both conditions equally.

Overall, our findings suggest that high-functioning autistic individuals might be able to (explicitly) attribute perceptual states of seeing *something*, but do not have a preference for an actress that can see through sunglasses or whose eyes are visible. Contrary to typical people (Teufel et al. 2010a), it could be that there is no bidirectional integration of physical cues and mental states, and consequently these social stimuli are less valued by autistic individuals.

### Limitations

The present results are a promising first step towards a better understanding of the mechanisms behind perceptual mentalizing and social motivation in ASC. However, we are aware that some aspects of our experimental design could be considered as limitations. First, the present study did not attempt to convince participants that they were seeing another person on a live video feed. We addressed perceptual mentalizing in a non-interactive way, such that we manipulated the belief about whether the actress could see *something* through the sunglasses, but not whether she could see *me*. There are three reasons that account for this decision in the context of our study. First, we wanted to distinguish perceptual mentalizing from audience effect, and test its effect on social motivation. Second, we aimed to replicate the setting of Dubey et al. (2015), which did not use a live stream. Finally, it is very hard to reliably make participants believe in a live stream, particularly when using repeated video-clips that participants can choose to view. The fact that participants with ASC seemed to distinguish between normal and opaque sunglasses implies that beliefs about what the actress can see may be able to impact on performance in the CAM task. However, this belief manipulation is limited in terms of ecological validity: testing the belief that someone can see *me* can yield findings with stronger implications for real world interactions.

Second, the empty sunglasses and paper-eyes sunglasses were designed to be matched for the presence of an eye-shape in the visual image, but not matched in the belief about what the actress can see. However, there were clear perceptual differences between these two conditions: the amount of visual and emotional information was lower for the paper-eyes sunglasses. Moreover, some participants (both typical and ASC) reported that they found the paper-eyes sunglasses somewhat scary. This could be a confounding factor influencing their choices, such that the lower preference for B−E+ when compared to B+E+ would not be due to the absence of beliefs, but rather due to preference for a full emotional display or to avoid a scary video-clip. The best way to create a stimulus with the appearance of eye-shapes but without the belief that others can see through might be a manipulation of a live/recorded video feed, but this is hard to achieve.

Third, the processing of stimuli where eyes are visible (empty and paper-eyes sunglasses) and stimuli where eyes are not visible (normal and opaque sunglasses) requires different cognitive inferences. In the first case, participants were able to determine if the actress could or could not see just from the visual image, with no need for instructions. However, in the latter case participants could only interpret the conditions in terms of the actress’ vision if they first took into account the colour of the frames, and then inferred its meaning. Thus, it could be that the need of this second order inference was too demanding to distinguish between normal and opaque sunglasses on a trial-by-trial basis. This limitation is a challenge, not just for the present study, but for all studies using a sunglasses- or goggles-belief manipulation. An alternative way to manipulate this belief could be the use of two different actresses and a cover story explaining that one of them is blind: instead of manipulating the colour of the frames, a simple symbol indicating whether the actress has full vision or is blind could be used instead.

A further possible limitation is that we do not have likeability ratings of the actress from the typical and autistic participants who took part in the study. It could be that the two groups differ in how much they liked the actress, which could be confounding the interpretation of our results. However, we think this is unlikely to happen because both groups preferred to view the actress with empty sunglasses (where face is completely visible) equally.

Finally, some of our results show marginal p values and medium effect sizes, and this means that they need to be interpreted with caution. Replication of these findings with bigger sample sizes and more power is needed draw decisive conclusions. For instance, a post-hoc power analysis with G*Power (Faul et al. 2009, 2007) showed that the study is underpowered to detect a 3-way interaction (power = 0.107). Testing a bigger sample size (i.e. increase power) could yield enough evidence to reliably find (or not) an interaction effect between Belief, Eyes and Group. This could clarify, for instance, whether the preference for the co-presence of physical and belief cues (B+E+) is exclusive to typical participants or not.

### Future Directions

These findings open up several avenues for further research. In particular, it would be interesting to take a second-person neuroscience perspective (Schilbach et al. 2013) and examine social motivation in situations where there is a genuine interaction, and actors can look back at participants. For example, in a setting similar to Myllyneva and Hietanen (2015), who use a liquid crystal window, do participants choose to look at an actor who can see them or one who cannot? Eye tracking data would also be valuable: Gobel et al. (2015) found that, when participants thought their faces would be later seen by the confederate in the videos, they made eye contact with the high-rank confederate less often, and eye contact with the low-rank confederate more often, than if they thought the confederate would not see them. We believe that the use of settings emulating real interactions is key to reliably assess the impact of beliefs and eye gaze on social motivation.

Interestingly, in our study one autistic participant reported that he would not mind eye gaze on a video-clip, but he would feel uncomfortable with it during real life interactions. As it is suggested by the second-person neuroscience approach, real social interactions require individuals to process, integrate and update a variety of dynamic social cues, and they need to do it spontaneously as the interaction develops. This is a much harder task than just interpreting the beliefs of someone in a video, where only eye gaze is modulated. Thus, the relevance of using such settings rather than those in which participants only *observe* the interacting partner spans beyond the study of social interactions *per se*, and can also have implications in clinical interventions. While many interventions for autistic individuals are directed at improving mentalizing (e.g. Social StoriesTM; Gray 1993) or learning the meaning of different facial expressions (Crissey 2007), we suggest that learning to integrate both beliefs and physical social cues might be key to enhance social motivation in autism. In line with the second-person neuroscience approach, the development of intervention strategies that use real-time social scenarios is crucial to understand how different types of social cues are integrated and how they are updated over time. Overall, the use of ecologically valid social stimuli will allow a thorough understanding of the difficulties that autistic people face in day-to-day interactions.

## Conclusions

The present study demonstrates that the processing of beliefs about whether others can see and the processing of eye-shapes can impact on measures of social motivation. This modulation is found in both the typical and autistic group, but in different directions: while the former prefers videos where the actress can see and eyes are visible, the latter shows no preference for these videos. This could be linked to differences in integrating physical cues and perceptual states of other people. Nevertheless, we acknowledge that better matched stimuli and ecologically valid settings are required, and hope that future studies will address these issues. Overall, these findings have implications for a better understanding of the cognitive mechanisms underlying social interactions, as well as the social difficulties faced by autistic people.

## Electronic supplementary material

Below is the link to the electronic supplementary material.

Supplementary material 1 (DOCX 45 KB)

## References

[CR1] Baron-Cohen S, Leslie AM, Frith U (1985). Does the autistic child have a “theory of mind”?. Cognition.

[CR2] Baron-Cohen S, Wheelwright S, Jolliffe T (1997). Is there a “language of the eyes”? Evidence from normal adults, and adults with autism or Asperger syndrome. Visual Cognition.

[CR3] Baron-Cohen S, Wheelwright S, Skinner R, Martin J, Clubley E (2001). The Autism-Spectrum Quotient (AQ): Evidence from Asperger Syndrome/high-functioning autism, males and females, scientists and mathematicians. Journal of Autism and Developmental Disorders.

[CR4] Cage E, Pellicano E, Shah P, Bird G (2013). Reputation management: Evidence for ability but reduced propensity in autism. Autism Research.

[CR5] Chevallier C, Grèzes J, Molesworth C, Berthoz S, Happé F (2011). Brief report: Selective social anhedonia in high functioning autism. Journal of Autism and Developmental Disorders.

[CR6] Chevallier C, Parish-Morris J, McVey A, Rump KM, Sasson NJ, Herrington JD, Schultz RT (2015). Measuring social attention and motivation in Autism spectrum disorder using eye-tracking: Stimulus type matters. Autism Research.

[CR7] Chevallier K, Troiani B, Schultz (2012). The social motivation theory of autism. Trends in Cognitive Neuroscience.

[CR8] Corden B, Chilvers R, Skuse D (2008). Avoidance of emotionally arousing stimuli predicts social-perceptual impairment in Asperger’s syndrome. Neuropsychologia.

[CR9] Crissey, P. (2007). Getting the message: Learning to read facial expressions.

[CR10] de Hamilton AF, Lind F (2016). Audience effects: What can they tell us about social neuroscience, theory of mind and autism?. Culture and Brain.

[CR11] Delmonte S, Balsters JH, McGrath J, Fitzgerald J, Brennan S, Fagan AJ, Gallagher L (2012). Social and monetary reward processing in autism spectrum disorders. Molecular Autism.

[CR12] Diagnostic and Statistical Manual of Mental Disorders 5th Ed. (2013).

[CR13] Dubey I, Ropar D, de Hamilton AF (2015). Measuring the value of social engagement in adults with and without autism. Molecular Autism.

[CR14] Emery NJ (2000). The eyes have it: The neuroethology, function and evolution of social gaze. Neuroscience and Biobehavioral Reviews.

[CR15] Faul F, Erdfelder E, Buchner A, Lang AG (2009). Statistical power analyses using G*Power 3.1: Tests for correlation and regression analyses. Behavior Research Methods.

[CR16] Faul F, Erdfelder E, Lang A-G, Buchner A (2007). G*Power: A flexible statistical power analysis program for the social, behavioral, and biomedical sciences. Behavior Research Methods.

[CR17] Furlanetto T, Becchio C, Samson D, Apperly I (2016). Altercentric interference in level 1 visual perspective taking reflects the ascription of mental states, not submentalizing. Journal of Experimental Psychology: Human Perception and Performance.

[CR18] Georgescu AL, Kuzmanovic B, Schilbach L, Tepest R, Kulbida R, Bente G, Vogeley K (2013). Neural correlates of “social gaze” processing in high-functioning autism under systematic variation of gaze duration. NeuroImage: Clinical.

[CR19] Gobel MS, Kim HS, Richardson DC (2015). The dual function of social gaze. Cognition.

[CR20] Gray CA (1993). Social Stories: Improving responses of students with autis with accurate social information. Focus on Autistic Behaviour and Other Developmental Disabilities.

[CR21] Hayden BY, Parikh PC, Deaner RO, Platt ML (2007). Economic principles motivating social attention in humans. Proceedings of The Royal Society. Biological Sciences.

[CR22] Izuma K, Matsumoto K, Camerer CF, Adolphs R (2011). Insensitivity to social reputation in autism. Proceedings of the National Academy of Sciences.

[CR23] Izuma K, Saito DN, Sadato N (2009). Processing of the incentive for social approval in the ventral striatum during charitable donation. Journal of Cognitive Neuroscience.

[CR24] Klin A, Jones W, Schultz R, Volkmar F, Cohen D (2002). Visual fixation patterns during viewing of naturalistic social situations as predictors of social competence in individuals with autism. Archives of Peneral Psychiatry.

[CR25] Kohls G, Schulte-Rüther M, Nehrkorn B, Müller K, Fink GR, Kamp-Becker I (2013). Reward system dysfunction in autism spectrum disorders. Social Cognitive and Affective Neuroscience.

[CR26] Laidlaw KEW, Foulsham T, Kuhn G, Kingstone A (2011). Potential social interactions are important to social attention. Proceedings of the National Academy of Sciences.

[CR27] Lord C, Risi S, Lambrecht L, Cook EH, Leventhal BL, Dilavore PC (2000). The Autism diagnostic observation schedule-generic: A standard measure of social and communication deficits associated with the spectrum of Autism. Journal of Autism and Developmental Disorders.

[CR28] Lord C, Rutter M, Lecouteur A (1994). Autism diagnostic interview—revised: A revised version of a diagnostic interview for carers of individuals with possible pervasive developmental disorders. Journal of Autism and Developmental Disorders.

[CR29] Myllyneva A, Hietanen JK (2015). There is more to eye contact than meets the eye. Cognition.

[CR30] Nuku P, Bekkering H (2008). Joint attention: Inferring what others perceive (and don’t perceive). Consciousness and Cognition.

[CR31] Schilbach L, Timmermans B, Reddy V, Costall A, Bente G, Schlicht T, Vogeley K (2013). Toward a second-person neuroscience. Behavioral and Brain Sciences.

[CR32] Scott-Van Zeeland AA, Dapretto M, Ghahremani DG, Poldrack RA, Bookheimer SY (2010). Reward processing in autism. Autism Research.

[CR33] Senju A, Hasegawa T (2005). Direct gaze captures visuospatial attention. Visual Cognition.

[CR34] Senju A, Johnson MH (2009). Atypical eye contact in autism: Models, mechanisms and development. Neuroscience Biobehavioural Reviews.

[CR35] Senju A, Johnson MH (2009). The eye contact effect: Mechanisms and development. Trends in Cognitive Sciences.

[CR36] Senju A, Southgate V, White S, Frith U (2009). Mindblind eyes: An absence of spontaneous theory of mind in Asperger syndrome. Science.

[CR37] Senju A, Tojo Y, Yaguchi K, Hasegawa T (2005). Deviant gaze processing in children with autism: An ERP study. Neuropsychologia.

[CR38] Shore DM, Heerey EA (2011). The value of genuine and polite smiles. Emotion.

[CR39] Stavropoulos KKM, Carver LJ (2014). Reward anticipation and processing of social versus nonsocial stimuli in children with and without autism spectrum disorders. Journal of Child Psychology and Psychiatry.

[CR40] Teufel C, Alexis DM, Clayton NS, Davis G (2010). Mental-state attribution drives rapid, reflexive gaze following. Attention, Perception & Psychophyics.

[CR41] Teufel C, Fletcher PC, Davis G (2010). Seeing other minds: attributed mental states influence perception. Trends in Cognitive Sciences.

[CR42] Teufel C, von dem Hagen E, Plaisted-Grant KC, Edmonds JJ, Ayorinde JO, Fletcher PC, Davis G (2013). What is social about social perception research?. Frontiers in Integrative Neuroscience.

[CR43] Wechsler D (1999). Wechsler abbreviated scale of intelligence (WASI).

[CR44] Wechsler D (1999). Wechsler adult intelligence scale (WAIS).

