# Supplementary material for: Do Beliefs About Whether Others Can See Modulate Social Seeking in Autism?
Source: J Autism Dev Disord. 2018 Oct 4;49(1):335–48. doi: 10.1007/s10803-018-3760-1 (PMC6331498; doi:10.1007/s10803-018-3760-1)
Supplement: Supplementary file 1 — Supplementary material 1 (DOCX 45 KB) [file 10803_2018_3760_MOESM1_ESM.docx]

**Supplementary Materials**

*S1. Stimuli ratings*

All 32 videos (8 possible actors for each of the 4 video categories) were rated on trustworthiness, friendliness and genuineness of the greeting to ensure that they were consistent. Eighteen typical adults (10 females and 8 males; mean age: 23.2±3) were recruited to perform this task. They were given written informed consent before doing the experiment, and were compensated £5 for their time and travel expenses. Participants sat in front of a computer and rated all the videos on a scale from 1 to 9 for each of the items (trustworthiness, friendliness and genuineness of the greeting); the duration of this task was approximately 20 minutes. The ratings for each model were pooled across the three items, and a two-way repeated measures ANOVA with Actor and Video Category as within-subjects factors was computed. Where the assumption of sphericity was violated, Greenhouse-Geisser estimate p-values were used. Results showed there was a main effect of Actor (F_3.523,186.721_=22.194; p<.001; n_p_^2^=.295), a main effect of Video Category (F_1.703,90.241_=40.929; p<.001; n_p_^2^=.436), and a tendency for an interaction between Actor and Video Category (F_13.227,701.049_=1.675; p=.06; n_p_^2^=.031). Post-hoc pairwise comparisons using Bonferroni’s adjustment showed that Actress E (female) and Actor G (male) were rated significantly higher (p<.01) and lower (p<.001) than all other actors, respectively. Similarly, we also found that empty sunglasses (B+E+) and paper-eyes sunglasses (B-E+) were rated significantly higher (p<.001) and lower (p<.01) than the other Video Categories, respectively. However, there was no difference in ratings between normal sunglasses (B+E-) and opaque sunglasses (B-E-). See Figure S1 for the results.

***
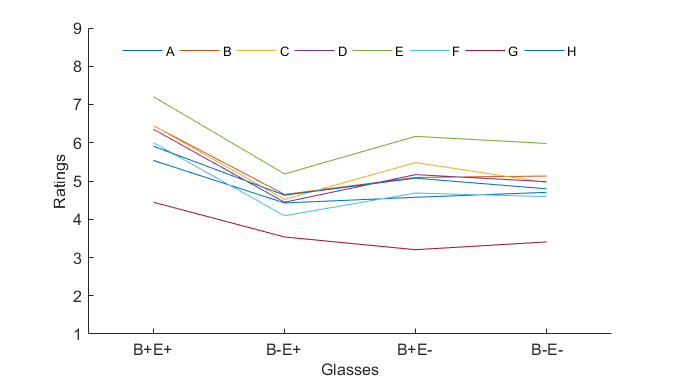
***

**Fig. S1** Mean rating score for each Actor (A-H) and Video Category.

*S2. Post-test questionnaire*

1. Can people see you through the normal glasses?

- Yes
- No

2. Can people see you through the fake eyes glasses?

- Yes
- No

3. Can people see you through the blue glasses?

- Yes
- No

4. Can people see you through the red glasses?

- Yes
- No

*S3. Choice score*

*S3.1. Model*

The choice score was modelled with MATLAB (8.5, MathWorks, 2015) using an algorithm that expanded upon the approach of Dubey and colleagues (Dubey et al., 2015). The algorithm assumes that each individual attaches a value V_x_ to each of the 4 video categories (x). Then on each trial the algorithm calculates the utility of choosing that video as a function of this value and the number of locks present on that trial. Thus, the utility for choosing x on trial i is U_i,x_ = V_x_ - L_i,x_ and this is calculated for each of the 4 video categories. The model then calculates the probability of choosing to view video x, normalised by the utilities of all the other videos. To obtain the optimal values V_x_ for each participant, the model calculates a cost function, which is the negative log sum of the probability of viewing the video that was actually chosen. We then use Matlab’s *fminsearch* function to find the values V_x_ which best predict the behavioural choices of this participant. We take these optimal values (*valout* below) to use for further analysis. The Matlab code for this is shown below:

%% set up for model optimisation

% vals is a 1x4 vector of the value of each choice

% locks is an nx4 matrix of the number of locks on each trial

% choice is an nx1 vector of the choices made by the participant

vals = [2,2,2,2]; % set starting point values (all videos worth 2 locks)

%% run the optimisation

[valout, cost] = fminsearch('modelchoice',vals, [], locks, choice);

% valout is a 1x4 vector representing the optimal values found for this participant

%%%%%%%%%%%%%%%%%%%%%%%%%%%%%%%%%%%%%%%%%%%%%%%%%%%%

%% Function modelchoice

function [cost] = modelchoice(vals, locks, choices)

util = repmat(vals,length(locks),1) - locks; % get utilities

sutil = sum(exp(util),2); % sum utilities

probpick = exp(util)./repmat(sutil,1,4); % get probability of picking each video

for i=1:length(probpick)

xpdf(i) = probpick(i,choices(i)); % keep the probability for the video

end % that the participant actually chose

%% Compute the cost function

cost = -sum(log(xpdf));

*S3.2. Analysis*

Descriptives for the choice score of each video category (23 typical, 22 ASC participants):

| Condition | Group | Mean | SD |
| --- | --- | --- | --- |
| Empty sunglasses  (B+E+) | Typical | 2.856 | .747 |
|  | Autism | 2.335 | .401 |
| Normal sunglasses  (B+E-) | Typical | 1.709 | .737 |
|  | Autism | 1.836 | .523 |
| Paper-eyes sunglasses  (B-E+) | Typical | 1.618 | .811 |
|  | Autism | 1.701 | .668 |
| Opaque sunglasses  (B-E-) | Typical | 1.701 | .837 |
|  | Autism | 2.071 | .404 |

Statistics for the Belief X Eyes X Group repeated measures ANOVA:

| Belief | main effect | | | *F*(1,43) = 23.9; *p* < .001^***^; n_p_^2^ = .358 |  |
| --- | --- | --- | --- | --- | --- |
| Eyes | main effect | | | *F*(1,43) = 4.76; *p* < .05^*^; n_p_^2^ = .100 |  |
| Group | main effect | | | *F*(1,43) = .502; *p* > .1; n_p_^2^ = .012 |  |
| Belief X Eyes | interaction effect | | | *F*(1,43) = 21.1; *p* < .001^***^; n_p_^2^ = .329 |  |
|  | B+: E+ vs. E- | | | *t*(47) = 5.09; *p* < .001^***^; d_z_ = .727 |  |
|  | B-: E+ vs. E- | | | *t*(47) = 1.17; *p* > .1; d_z_ = .167 |  |
|  | E+: B+ vs. B- | | | *t*(47) = 6.04; *p* < .001^***^; d_z_ = .863 |  |
|  | E-: B+ vs. B- | | | *t*(47) = .891; *p* > .1; d_z_ = .127 |  |
| Belief X Group | | interaction effect | *F*(1,43) = 6.36; *p* < .05^*;^ n_p_^2^ = .129 | | |
|  |  | B+: typ vs. aut | *t*(47) = 2.16; *p* < .05^*^; d_z_ = .309 | | |
|  |  | B-: typ vs. aut | *t*(47) = 2.77; *p* < .01^**^; d_z_ = .395 | | |
|  |  | Typ: B+ vs. B- | *t*(47) = 5.28; *p* < .001^***^; d_z_ = .754 | | |
|  |  | Aut: B+ vs. B- | *t*(47) = 1.66; *p* > .1; d_z_ = .237 | | |
| Eyes X Group | | interaction effect | *F*(1,43) = 2.91; *p* = .095^+^; n_p_^2^ = .064 | | |
|  |  | E+: typ vs. aut | *t*(47) = 1.68; *p* = .099^+^; d_z_ = .241 | | |
|  |  | E-: typ vs. aut | *t*(47) = 1.69; *p* = .097^+^; d_z_ = .242 | | |
|  |  | Typ: E+ vs. E- | *t*(47) = 2.79; *p* < .01^**^; d_z_ = .399 | | |
|  |  | Aut: E+ vs. E- | *t*(47) = .332; *p* > .1; d_z_ = .047 | | |
| Belief X Eyes X Group | | interaction effect | *F*(1,43) = .620; *p* > .1; n_p_^2^ = .014 | | |

*S4. Number of locks analysis*

Descriptives for the proportion of times (0-1) each number of locks was chosen (25 typical, 24 ASC participants):

| Locks | Group | Mean | SD |
| --- | --- | --- | --- |
| 1 | Typical | .415 | .136 |
|  | Autism | .400 | .136 |
| 2 | Typical | .317 | .047 |
|  | Autism | .319 | .053 |
| 3 | Typical | .268 | .123 |
|  | Autism | .281 | .104 |

Statistics for the Locks X Group repeated measures ANOVA:

| Locks | main effect | *F*(2,94) = 13.2; *p* < .001^***^; n_p_^2^ = .220 |
| --- | --- | --- |
|  | 1 vs. 2 | *t*(47) = 3.71; *p* < .01^**^; d_z_ = .530 |
|  | 1 vs. 3 | *t*(47) = 3.8; *p* < .01^**^; d_z_ = .543 |
|  | 2 vs. 3 | *t*(47) = 2.75; *p* < .05^*^; d_z_ = .393 |
| Group | main effect | ^a^*F*(1,47) = . ; *p* = . ; n_p_^2^ = 1 |
| Locks X Group | interaction effect | *F*(2,94) = .140; *p* > .1; n_p_^2^ = .003 |

^a^Both groups have same means
